# Factors affecting the presence of depression, anxiety disorders, and suicidal ideation in patients attending primary health care service in Lithuania

**DOI:** 10.3109/02813432.2013.873604

**Published:** 2014-03

**Authors:** Robertas Bunevicius, Vilma Liaugaudaite, Jurate Peceliuniene, Nijole Raskauskiene, Adomas Bunevicius, Narseta Mickuviene

**Affiliations:** ^1^Behavioral Medicine Institute of the Lithuanian University of Health Sciences, Palanga, Lithuania; ^2^Laboratory of Clinical Research, Institute of Neurosciences, Lithuanian University of Health Sciences, Kaunas, Lithuania

**Keywords:** Anxiety disorder, depression, general practice, Lithuania, primary care, recognition, suicidal ideation

## Abstract

*Objective*. The aim of this study was to establish prevalence, recognition, and risk factors for mental disorders and suicidal ideation in PC patients. *Design*. A cross-sectional survey based on standard mental health evaluation. *Setting*. Lithuanian primary care. *Subjects*. 998 patients from four urban PC clinics. *Main outcome measures*. Current mental disorders and suicidal ideation assessed using the Mini International Neuropsychiatric Interview (MINI). *Results*. According to the MINI, 27% of patients were diagnosed with at least one current mental disorder. The most common mental disorders were generalized anxiety disorder (18%) and major depressive episode (MDE) (15%), followed by social phobia (3%), panic disorder (3%), and post-traumatic stress disorder (2%). Some 6% of patients reported suicidal ideation. About 70% of patients with current mental disorder had no documented psychiatric diagnosis and about 60% received no psychiatric treatment. Greater adjusted odds for current MDE were associated with being widowed or divorced patients (odds ratio, OR = 1.8, 95% CI 1.2–2.8) and with lower education (OR = 1.6, 95% CI 1.1–2.3), while greater adjusted odds for any current anxiety disorder were found for women (OR = 1.9, 95% CI 1.3–2.8) and for patients with documented insomnia (OR = 2.2, 95% CI 1.2–4.2). Suicidal ideation was independently associated with use of antidepressants (OR = 5.4, 95% CI 1.7–16.9), with current MDE (OR = 2.9, 95% CI 1.5–5.8), and with excessive alcohol consumption (OR = 2.0, 95% CI 1.1–3.8). *Conclusions*. Depression, anxiety disorders, and suicidal ideation are prevalent but poorly recognized among PC patients. The presence of current MDE is independently associated with marital status and with lower education, while current anxiety disorder is associated with female gender and insomnia. Suicidal ideation is associated with current MDE, and with antidepressants and alcohol use.

- Primary care (PC) services have a key role in provision of mental health for patients with mild to moderate mental disorders.
- Mental health issues are prevalent among PC patients but are poorly identified and managed.
- Presence of depression is associated with loss of spouse and lower education; presence of anxiety disorder is associated with female gender and insomnia.
- Suicidal ideation is associated with current depression, antidepressant use and excessive alcohol consumption.

## Introduction

Depression and anxiety disorders are highly prevalent in the primary care (PC) patient population and are associated with increased risk for medical illness and with decreased level of functioning [1]. The PC setting is expected to be the first contact point for people suffering from mental health problems [2]. Notwithstanding, mental disorders remain poorly recognized and managed in the PC setting mainly because PC health providers face significant time constraints, and receive limited training with regard to identification and management of mental health issues [3]. Existing evidence suggests that training of PC physicians to identify and to treat mood disorders may significantly improve individual clinical and mental health outcomes [4].

A substantial body of evidence suggests that the outcome of medical illness can be improved if comorbid mental disorders are recognized in a timely fashion and effectively treated [5]. In addition, untreated psychiatric disorders are associated with increased rates of suicidal behaviour and psychoactive substance use [6]. Lithuania has the highest suicide rate in Europe [7]. It has been shown that a majority of suicidal patients have been in close contact with PC services recently prior to suicide attempts [8]. Thus, identification of risk factors for suicidal ideation in the PC patient population can potentially improve suicide awareness and prevention.

We aimed to evaluate the prevalence, management, and risk factors of depression, anxiety disorders, and suicidal ideation in PC patients.

## Material and methods

### Study sample and procedure

The study protocol and informed consent form were approved by the Lithuanian Bioethics Committee. Each patient signed an informed consent form. The study was performed in four PC settings in two major cities of Lithuania. In each PC setting, during a four-week period, consecutive patients attending their family practitioner were invited to participate in this cross-sectional study. Recruitment of patients lasted four hours each day. There were no exclusion criteria but only subjects aged 18 or older were invited into the study. Patients who were excluded from the study did not differ from the patients who were studied in terms of gender, age, marital status, and education (p > 0.05).

After visiting their family practitioner, patients were interviewed by a trained physician for current psychiatric diagnoses, suicidal ideation, and excessive alcohol consumption by means of the Mini International Neuropsychiatric Interview (MINI) [9] as well as for socio-demographic status and for current psychiatric treatment. Information regarding documented psychiatric diagnosis was collected from medical records in terms of ICD-10 codes.

### Methods

The MINI is a well-validated, standardized, and structured diagnostic interview that provides an evaluation of psychiatric diagnoses according to the American Psychiatric Association's Diagnostic and Statistical Manual for Mental Disorders, Fourth edition, text revision (DSM-IV-TR) criteria [10]. The MINI is a validated instrument for evaluation of mental disorders in psychiatric populations and in general medical populations including PC patients [11]. We used MINI models that cover current diagnoses of major depressive episode (MDE), post- traumatic stress disorder (PTSD), panic disorder, social phobia, and generalized anxiety disorder (GAD). According to the MINI suicidal ideation screening items, patients were considered as having a suicidal ideation if during the past month they had thoughts that they would be better off dead or wished that they were dead; or wanted to harm himself or herself; or thought about suicide; or had a suicide plan; or attempted suicide. Patients were evaluated for excessive alcohol use by using the following MINI screening question: “In the past 12 months, have you had three or more alcoholic drinks within a three-hour period on three or more occasions?” Administration of these models of the MINI takes from three to 20 minutes, depending on the presence and complexity of psychiatric diagnoses.

### Statistics

The Statistical Package for Social Sciences (SPSS 15) for Windows program was used for statistical analysis. The prevalence rates are presented as percentages and 95% confidence interval (CI). Differences in prevalence between documented psychiatric diagnoses and current mental disorders established by the MINI were analysed by employing the chi-squared test. All tests were two sided and statistical significance was assumed when the p-value was < 0.05.

Univariate and multivariate (enter method of analysis) binary logistic regression analyses were employed to assess the association of MDE, anxiety disorders, and suicidal ideation with socio-demographic and clinical factors.

## Results

In total 1170 patients were approached. However, 160 (14%) patients declined to participate, and 12 (1%) patients were excluded from further analysis due to incomplete data. Therefore, the final study sample included 998 patients. The study patients were predominantly women (68%), were married (60%), and had higher than secondary education (67%). The majority of patients did not have a documented psychiatric diagnosis (85%), did not consult a psychiatrist (79%), and did not receive psychiatric treatment (80%) (Table I).

**Table I.** Demographic and clinical characteristics of study sample (n = 998).

Characteristics	
Age, mean (SD); range, years	50 (19); 18–89
Gender, n (%):	
Women	678 (68)
Men	320 (32)
Marital status, n (%):	
Married/unmarried	778 (78)
Divorced/widowed	220 (22)
Education, n (%):	
Secondary or below	334 (33)
Any postsecondary	664 (67)
Main reason for the visit, n (%):	
Consultations	822 (82)
Administrative reason	176 (18)
Documented psychiatric diagnosis, n (%):	
Depressive disorder	45 (4.5)
Anxiety disorder	30 (3)
Insomnia	50 (5)
Psychotic disorder	12 (1.2)
Other	13 (1.3)
No psychiatric diagnosis	848 (85)
Documented psychiatric consultation, n (%)	205 (20)
Current use of psychiatric medication, n (%):	
Antidepressants	23 (2.3)
Anxiolytics	143 (14)
Antipsychotics	11 (1.1)
No psychiatric medication	793 (80)

According to the MINI interview, 27% of patients met the diagnostic criteria for at least one current mental disorder (Table II), 21% of patients met the criteria for at least one anxiety disorder, and 15% of patients for MDE. GAD was the most common anxiety disorder (18%), followed by social phobia (3%), panic disorder (3%), and PTSD (2%). Suicidal ideation was identified in 6% of patients and 35% of patients were considered as having excessive alcohol consumption. The prevalence of any mental disorders, MDE, GAD, and panic disorder was significantly greater in women. On the other hand, excessive alcohol consumption was more prevalent in men. The majority of patients with current MINI diagnoses did not have a documented psychiatric diagnosis (71%) (Table II). More than half of patients with current MDE or current anxiety disorder did not receive psychiatric treatment (62% and 65% respectively) (Table III).

**Table II.** Prevalence of current mental disorders established by the Mini International Neuropsychiatric Interview.

Current mental disorder	No. with disorder	Prevalence % (95% CI)		
	(Men/women)	All (n = 998)	Men (n = 320)	Women (n = 678)
Major depressive episode^1^	152 (34/118)	15 (13–17)	10 (7–14)	17 (14–20)
Any anxiety disorder	212 (45/167)	21 (19–24)	14 (11–18)	25 (21–28)
Generalized anxiety disorder^1^	180 (37/142)	18 (16–20)	12 (8–15)	21 (18–24)
Social phobia	32 (10/22)	3 (2–4)	3 (1–5)	3 (2–4)
Panic disorder^1^	27 (3/24)	3 (2–4)	1 (0.1–2)	3 (2–5)
Post-traumatic stress disorder	23 (4/19)	2 (1–3)	1 (0.1–2)	3 (2–4)
Any mental disorder^1^	266 (60/206)	27 (24–29)	19 (14–23)	30 (27–34)
Suicidal ideation	61 (16/45)	6 (5–8)	5 (3–7)	7 (5–8)
Excessive alcohol consumption^2^	348 (174/174)	35 (32–38)	54 (49–60)	26 (22–29)

Notes: ^1^Women > men, p < 0.05; ^2^men > women, p < 0.05.

**Table III.** Documented psychiatric diagnoses and treatments in patients with current mental disorders established by the Mini International Neuropsychiatric Interview.

Documented psychiatric diagnosis, n (%)	Current MINI diagnoses of mental disorders	
	Major depressive episode (n = 152)	Any anxiety disorder^1^ (n = 212)
Depressive disorder	16 (11)	23 (11)
Any anxiety disorder	6 (4)	10 (5)
Insomnia	11 (7)	18 (8)
Psychotic disorder	6 (4)	6 (3)
Other	3 (2)	6 (3)
No psychiatric diagnosis	110 (71)	149 (70)
Psychiatric treatment:		
Antipsychotics	4 (3)	4 (2)
Antidepressants	5 (3)	15 (7)
Anxiolytics	39 (26)	75 (35)
No psychiatric treatment	85 (62)	138 (65)

Note: ^1^At least one of four anxiety disorders: post-traumatic stress disorder, panic disorder, social phobia, or generalized anxiety disorder.

Greater adjusted odds for current MDE were associated with being widowed or divorced (OR = 1.8, 95% CI 1.2–2.8, p = 0.007) and with lower education (OR = 1.6, 95% CI 1.1–2.3, p = 0.013) (Table IV). Interestingly, excessive alcohol use was associated with reduced adjusted odds for current MDE (OR = 0.6, 95% CI 0.4–0.9, p = 0.033). Greater adjusted odds for any current anxiety disorder were found for women (OR = 1.9, 95% CI 1.3–2.8, p = 0.010) and for patients with documented insomnia (OR = 2.2, 95% CI 1.2–4.2, p = 0.012).

**Table IV.** Factors affecting the presence of current mental disorders established by the Mini International Neuropsychiatric Interview.

	OR (95% CI)			
	Major depressive episode		Any anxiety disorder^1^	
	Univariate	Adjusted^2^	Univariate	Adjusted^2^
Age, years	**1.01 (1.00–1.02)**	0.99 (0.98–1.01)	0.99 (0.98–1.0)	0.99 (0.98–1.01)
Female gender	**1.8 (1.2–2.7)**	1.4 (0.9–2.1)	**1.9 (1.4–2.9)**	**1.9 (1.3–2.8)**
Widowed or divorced^3^	**2.1 (1.4–3.0)**	**1.8 (1.2–2.8)**	1.5 (0.9–2.3)	1.03 (0.7–1.5)
Education, secondary or below^4^	**1.6 (1.1–2.3)**	**1.6 (1.1–2.3)**	1.3 (0.9–1.8)	1.3 (0.9–1.8)
Documented insomnia	1.6 (0.8–3.2)	1.4 (0.7–2.9)	**2.2 (1.2–3.9)**	**2.2 (1.2–4.2)**
Excessive alcohol consumption	**0.5 (0.3–0.8)**	**0.6 (0.4–0.9)**	**0.7 (0.5–0.9)**	0.8 (0.6–1.2)

Notes: ^1^At least one of four anxiety disorders: post-traumatic stress disorder, panic disorder, social phobia, and generalized anxiety disorder; ^2^for all variables in the table; ^3^vs. married or unmarried; ^4^vs. any postsecondary. OR, odds ratios in bold differ significantly (p < 0.05) from 1.0.

Table V shows that increased adjusted odds for suicidal ideation were associated with current MDE (OR = 2.9, 95% CI 1.5–5.8, p = 0.003) and with use of antidepressants (OR = 5.4, 95% CI 1.7–16.9, p = 0.004). In addition, excessive alcohol consumption was not associated with suicidal ideation in the univariate model, but reached statistical significance in the adjusted model (OR = 2.0, 95% CI 1.1–3.8, p = 0.026).

**Table V.** Factors affecting the presence of suicidal ideation.

	OR (95% CI)	
	Univariate	Adjusted^1^
Age, years	1.03 (0.98–1.02)	1.0 (0.98–1.02)
Female gender	1.3 (0.7–2.4)	1.5 (0.8–2.9)
Widowed or divorced^2^	0.9 (0.4–2.1)	0.9 (0.4–1.7)
Education, secondary or below^3^	1.1 (0.7–1.9)	1.2 (0.7–2.1)
Current major depressive episode	**3.7 (2.2–6.6)**	**2.9 (1.5–5.8)**
Any current anxiety disorder^4^	**3.0 (1.8–5.1)**	1.6 (0.8–3.0)
Excessive alcohol consumption	1.3 (0.8–2.2)	**2.0 (1.1–3.8)**
Psychiatric medication use:		
Antipsychotics	1.9 (0.2–15.5)	1.4 (0.2–12.8)
Antidepressants	**5.4 (1.9–15.2)**	**5.4 (1.7–16.9)**
Anxiolytics	**2.2 (1.2–4.2)**	1.9 (0.9–3.9)

Notes: ^1^For all variables in the table; ^2^vs. married or unmarried; ^3^vs. any postsecondary; ^4^at least one of four anxiety disorders: post-traumatic stress disorder, panic disorder, social phobia, and generalized anxiety disorder. OR, odds ratios in bold differ significantly (p < 0.05) from 1.0.

## Discussion

Our study confirmed previous findings that depression and anxiety disorders as well as suicidal ideation are prevalent but poorly recognized and managed in PC settings. We found that the presence of MDE was independently associated with lower education, with loss of spouse, and with less extensive alcohol consumption. The presence of anxiety disorder was associated with female gender and with documented insomnia. Suicidal ideation was associated with current MDE, with use of antidepressants, and with excessive alcohol consumption.

The WHO study found that PC physicians diagnosed only 39% of current depression cases [12]. Similarly, another study found that two-thirds of individuals with depression remained undiagnosed in PC settings [13]. Our study yielded similar results: we found that one-third of patients with current depression or anxiety disorder had a documented psychiatric diagnosis and received treatment. Reports from the Netherlands and the USA [14] have shown that less than 40% of depressed individuals were receiving treatment for depression and only a small proportion were adequately treated in PC settings, primarily because of the failure to recognize depression. The major obstacle for such failure is that PC physicians often overestimate somatic complaints and pay less attention to psychological symptoms [15]. The problem of under-detection and under-treatment of psychiatric morbidity in PC has not been solved despite increasing evidence that comorbid depression and anxiety disorders as well as suicidal ideation have a negative impact on the course and outcomes of medical conditions [16–18]. Therefore, global and local initiatives increasing awareness, recognition, and management of psychiatric disorders in the PC setting are urgently needed.

We found that loss of spouse, lower education status, and lower alcohol consumption were independent risk factors for depression, whereas female gender together with documented insomnia was associated with increased odds for anxiety disorder. Poor social support and female gender are well- recognized risk factors for psychiatric disorders [19]. With regard to the association of insomnia with anxiety disorder, other studies indicate that insomnia is more prevalent in patients with anxiety disorder and insomnia is considered a risk factor for anxiety disorder [20]. In our study, excessive alcohol consumption was associated with reduced odds for depression. It should be recalled that the MINI screening item for alcohol consumption uses a very low threshold; therefore, a large proportion of patients with moderate alcohol use were categorized as excessive alcohol users. A U-shaped association of alcohol consumption with depression was previously reported, since relative to abstinence and heavy alcohol consumption, moderate alcohol consumption was demonstrated to be protective against depression [21] and only heavy drinking or abstinence was associated with an increased risk of experiencing mental disorders [22]. In a similar way, a recent multi-centre study showed that episodic drinking was less common in people with existing MDE as compared with non-depressed subjects [23].

We found that the most important risk factors for suicidal ideation were current use of anti-depressant medication, current MDE, and extensive alcohol consumption, which corresponds with the data from other countries [24]. Others have estimated that up to 71% of MDE patients express current suicidal ideation [25], about one in 10 of depressed patients attempt suicide, and about 70% of all suicides revolve around depressive or anxiety disorder [26]. Current use of antidepressants is a well-established risk factor for suicidal ideation [27]. For example, a study from the Netherlands found that 60% of patients who committed or attempted suicide were diagnosed as depressed, of whom 91% were treated with antidepressants [28]. Therefore, identification of depression should be followed by aggressive management, and suicidal ideation should be carefully monitored in depressed patients receiving antidepressant treatment.

The finding that excessive alcohol consumption doubled the risk for suicide ideation is not surprising, since approximately one-third of suicides are associated with alcohol use and up to 10% of people who are dependent on alcohol end their life by committing suicide [29]. Therefore, PC patients with excessive alcohol consumption should receive proper counselling and should be asked about suicidal ideation.

Some country-specific factors may play a role in recognition and management of mental disorders and suicidality in PC settings. Currently, two parallel and separate PC systems coexist in Lithuania. PC health centres employ family practitioners and are expected to cover the majority of medical problems whereas mental health problems are assigned to primary mental health centres where psychiatry teams are employed. Such a system may contribute to poor recognition and management of mental disorders by family practitioners as they may assign mental health issues to primary mental health care services [30].

The large sample and participation rate of consecutive PC patients and the use of standard and structured diagnostic instruments are major strengths of our study. On the other hand, the cross-sectional design prevented us from addressing a causal relationship between suicidal ideation, psychiatric disorders, excessive alcohol consumption, and treatment of psychiatric disorders and is the major limitation of the study. Specific features of the Lithuanian health care system limit the generalizability of our findings.

## Conclusions

Depression, anxiety disorders, and suicidal ideation are prevalent among patients attending PC clinics but are poorly identified and managed by family practitioners. The presence of current depression is independently associated with marital status and with lower education, while current anxiety disorders are independently associated with female gender and with documented insomnia. Suicidal ideation is associated with current MDE, with antidepressant use, and with excessive alcohol consumption.

## References

[1] Ansseau M, Dierick M, Buntinkx F, Cnockaert P, De Smedt J, Van Den Haute M, Vander Mijnsbrugge D (2004). High prevalence of mental disorders in primary care. J Affect Disord.

[2] King M, Nazareth I, Levy G, Walker C, Morris R, Weich S (2008). Prevalence of common mental disorders in general practice attendees across Europe. Br J Psychiatry.

[3] Ostergaard SD, Foldager L (2011). The association between physical illness and major depressive episodes in general practice. Acta Psychiatr Scand.

[4] Bunevicius A, Peceliuniene J, Mickuviene N, Valius L, Bunevicius R (2007). Screening for depression and anxiety disorders in primary care patients. Depress Anxiety.

[5] Simon GE, Von Korff M, Lin E (2005). Clinical and functional outcomes of depression treatment in patients with and without chronic medical illness. Psychol Med.

[6] Kessler RC, Borges G, Walters EE (1999). Prevalence of and risk factors for lifetime suicide attempts in the National Comorbidity Survey. Arch Gen Psychiatry.

[7] World Health Organization Suicide rates (per 100,000) by country, year and sex, 2011. http://www.who.int/mental_health/prevention/suicide_rates/en/2013.

[8] Wasserman D, Rihmer Z, Rujescu D, Sarchiapone M, Sokolowski M, Titelman D (2012). The European Psychiatric Association (EPA) guidance on suicide treatment and prevention. Eur Psychiatry.

[9] Sheehan DV, Lecrubier Y, Sheehan KH, Amorim P, Janavs J, Weiller E (1998). The Mini-International Neuropsychiatric Interview (M.I.N.I.): the development and validation of a structured diagnostic psychiatric interview for DSM-IV and ICD-10. J Clin Psychiatry.

[10] American Psychiatric Association (2000). Diagnostic and Statistical Manual of Mental Disorders. 4th ed., Text Revision.

[11] Bunevicius R, Velickiene D, Prange AJ (2005). Mood and anxiety disorders in women with treated hyperthyroidism and ophthalmopathy caused by Graves’ disease. Gen Hosp Psychiatry.

[12] Celano CM, Huffman JC (2011). Depression and cardiac disease: A review. Cardiol Rev.

[13] Ani C, Bazargan M, Hindman D, Bell D, Farooq MA, Akhanjee L (2008). Depression symptomatology and diagnosis: Discordance between patients and physicians in primary care settings. BMC Fam Pract.

[14] Kamphuis MH, Stegenga BT, Zuithoff NP, King M, Nazareth I, de Wit NJ (2012). Does recognition of depression in primary care affect outcome?. The PREDICT-NL study. Fam Pract.

[15] Arnow BA (2004). Depression and physical symptoms: The mind–body connection. J Clin Psychiatry.

[16] Christensen KS, Sokolowski I, Olesen F (2011). Case-finding and risk-group screening for depression in primary care. Scand J Prim Health Care.

[17] Ostergaard SD, Foldager L, Allgulander C, Dahl AA, Huuhtanen MT, Rasmussen I (2010). Psychiatric caseness is a marker of major depressive episodes in general practice. Scand J Prim Health Care.

[18] Lyness JM, Heo M, Datto CJ, Ten Have TR, Katz IR, Drayer R (2006). Outcomes of minor and subsyndromal depression among elderly patients in primary care settings. Ann Intern Med.

[19] Dalla C, Pitychoutis PM, Kokras N, Papadopoulou-Daifoti Z (2011). Sex differences in response to stress and expression of depressive-like behaviours in the rat. Curr Top Behav Neurosci.

[20] Breslau N, Roth T, Rosenthal L, Andreski P (1996). Sleep disturbance and psychiatric disorders: A longitudinal epidemiological study of young adults. Biol Psychiatry.

[21] Gea A, Martinez-Gonzalez MA, Toledo E, Sanchez-Villegas A, Bes-Rastrollo M, Nuñez-Cordoba JM (2012). A longitudinal assessment of alcohol intake and incident depression: The SUN project. BMC Public Health.

[22] Skogen JC, Harvey SB, Henderson M, Stordal E, Mykletun A (2009). Anxiety and depression among abstainers and low-level alcohol consumers. The Nord-Trøndelag Health Study. Addiction.

[23] Nazareth I, Walker C, Ridolfi A, Aluoja A, Bellon J, Geerlings M (2011). Heavy episodic drinking in Europe: A cross section study in primary care in six European countries. Alcohol Alcohol.

[24] Pilowsky DJ, Olfson M, Gameroff MJ, Wickramaratne P, Blanco C, Feder A (2006). Panic disorder and suicidal ideation in primary care. Depress Anxiety.

[25] Inagaki M, Ohtsuki T, Yonemoto N, Oikawa Y, Kurosawa M, Muramatsu K (2013). Prevalence of depression among outpatients visiting a general internal medicine polyclinic in rural Japan. Gen Hosp Psychiatry.

[26] Kessler RC, Borges G, Walters EE (1999). Prevalence of and risk factors for lifetime suicide attempts in the National Comorbidity Survey. Arch Gen Psychiatry.

[27] Almeida OP, Draper B, Snowdon J, Lautenschlager NT, Pirkis J, Byrne G (2012). Factors associated with suicidal thoughts in a large community study of older adults. Br J Psychiatry.

[28] Marquet RL, Bartelds AI, Kerkhof AJ, Schellevis FG, van der Zee J (2005). The epidemiology of suicide and attempted suicide in Dutch General Practice 1983–2003. BMC Fam Pract.

[29] WHO (World Health Organization), Department of Mental Health (2000). Preventing suicide: A resource for primary health care workers.

[30] Stein MB, Roy-Byrne PP, Craske MG, Bystritsky A, Sullivan G, Pyne JM (2005). Functional impact and health utility of anxiety disorders in primary care outpatients. Med Care.

